# Accessibility of Medicare Diabetes Prevention Programs and Variation by State, Race, and Ethnicity

**DOI:** 10.1001/jamanetworkopen.2021.28797

**Published:** 2021-10-08

**Authors:** Alice Yan, Zhuo Chen, MinQi Wang, Carlos E. Mendez, Leonard E. Egede

**Affiliations:** 1 Center for Advancing Population Science, Division of Internal Medicine, Department of Medicine, Medical College of Wisconsin, Milwaukee; 2 Department of Health Policy and Management, College of Public Health, University of Georgia, Athens; 3 School of Economics, Faculty of Humanities and Social Sciences, University of Nottingham Ningbo China, Zhejiang, China; 4 Department of Behavioral and Community Health, School of Public Health, University of Maryland, College Park; 5 Clement J. Zablocki VA Medical Center, Wisconsin, Milwaukee; 6 Division of Internal Medicine, Department of Medicine, Medical College of Wisconsin, Milwaukee

## Abstract

This cross-sectional study examines the accessibility of the Medicare Diabetes Prevention Program and investigates whether there are disparities in access among racial and ethnic minority beneficiaries at the state level.

## Introduction

Medicare implemented the Medicare Diabetes Prevention Program (MDPP) to reimburse all eligible beneficiaries at risk of diabetes or with prediabetes for the DPP since April 1, 2018. Although it has been 3 years since the MDPP performance period began and there is a need for broad uptake to meet the needs of 61 million Medicare beneficiaries^1,2^ (eg, 48.3% of whom are estimated to have prediabetes),^3^ there is limited data on access to and uptake of the MDPP in the United States. The disproportionate burden of obesity and type 2 diabetes on racial and ethnic minority communities signals an urgent need to accelerate the implementation and coordination of the MDPP. This study evaluates the accessibility of the MDPP and investigates whether there are disparities in access among racial and ethnic minority beneficiaries at the state level.

## Methods

This cross-sectional study used data from the Centers for Medicare & Medicaid Services (CMS) registry of Medicare-enrolled MDPP suppliers list as of March 26, 2021,^4^ publicly available 2019 Medicare Beneficiary Enrollment population data by states and the District of Columbia (DC) and by race and ethnicity, and diabetes prevalence data at the state level from the 2019 Behavioral Risk Factor Surveillance System.^5^ Informed consent was waived because data were publicly available and deidentified. The study was deemed exempt by the institutional review board at the Medical College of Wisconsin. This study followed the Strengthening the Reporting of Observational Studies in Epidemiology (STROBE) reporting guideline.

Individuals self-identified their race for each of the data lists. Only populations of Black or African American people, Hispanic/Latinx people, and non-Hispanic White people were included. Given that a single supplier can offer MDPP at multiple sites, site availability was the primary indicator of MDPP access.

First, we linked the MDPP supplier data with Medicare Beneficiary Enrollment data by state and by race and ethnicity. Second, we examined the type of suppliers and evaluated access, which was defined as the number of Medicare beneficiaries per MDPP site for each state. Third, to identify potential gaps in MDPP access by diabetes burden and by race and ethnicity across states, we used geographic information system to map percentages of African American individuals or Hispanic and Latinx individuals enrolled in Medicare among the total population across states. One-way analysis of variance compared the differences in African American individuals or Hispanic and Latinx individuals per MDPP site across states with 3 levels of Medicare enrollment (ie, ≤10%, 10-20%, and ≥20%) using STATA version 16 (StataCorp). Statistical tests were 2-tailed, and significance was set at *P* < .05. Data analysis was conducted from May to July 2021.

## Results

There were 244 unique suppliers that offered the MDPP across 940 sites in 46 states and Washington DC (Table). With approximately 61 million Medicare beneficiaries in 2020, this equates to 1.5 sites per 100 000 Medicare beneficiaries. Two states (ie, Connecticut and New Mexico) and Washington DC had only 1 site for 640 932 beneficiaries, which is equivalent to 213 644 Medicare beneficiaries per site.

**Table.** Number and Frequency of Medicare Diabetes Prevention Program Suppliers by Organization Type as of March 2021

Organization type	No. (%) (N = 244)
American Indian services	4 (0.4)
Area agencies on aging and disabilities	9 (1.0)
Community health center	6 (0.6)
Community organization	2 (0.2)
Community-based social service agency	4 (0.4)
Community/retail pharmacy	4 (0.4)
Consumer service company	2 (0.2)
Drug wholesalers industry	3 (0.3)
Federally qualified health center (nonprofit)	11 (1.2)
For-profit company	1 (0.1)
Foundation	163 (17.3)
Health care company	10 (1.1)
Health coaching company	1 (0.1)
Health educator provider	13 (1.4)
Health plan	24 (2.6)
Health system	368 (39.1)
Health, wellness, and fitness company	14 (1.5)
Home health agency	2 (0.2)
Nonprofit organization	9 (1.0)
Physical therapy	4 (0.4)
Private practice	1 (0.1)
Public health agencies	67 (7.1)
Supermarkets	2 (0.2)
University	23 (2.4)
Wellness and fitness center	5 (0.5)
YMCA	188 (20.0)
Total	940

Abbreviation: YMCA, Young Men’s Christian Association.

There was a significant difference in African American beneficiaries per site (*F*_2, 44_ = 38.31, *P* < .001) across 3 state categories of enrollment of African American individuals. Mississippi, Louisiana, Maryland, Georgia, and Washington DC had an African American Medicare beneficiary population 20% or more, and Mississippi and Louisiana also bear some of the nation’s highest type 2 diabetes burdens (14.8% and 12.8%, respectively) (Figure). However, except for Maryland, which had 30 sites, the remaining states only had 1 to 3 sites. Similarly, Hispanic and Latinx beneficiaries per site differed significantly (*F*_2, 44_ = 63.86, *P* < .001) across 3 state categories of enrollment of Hispanic or Latinx individuals. Although New Mexico has a Medicare beneficiary population of Hispanic or Latinx individuals of 20% or more, it only had 1 MDPP site (Figure).

**Figure.**
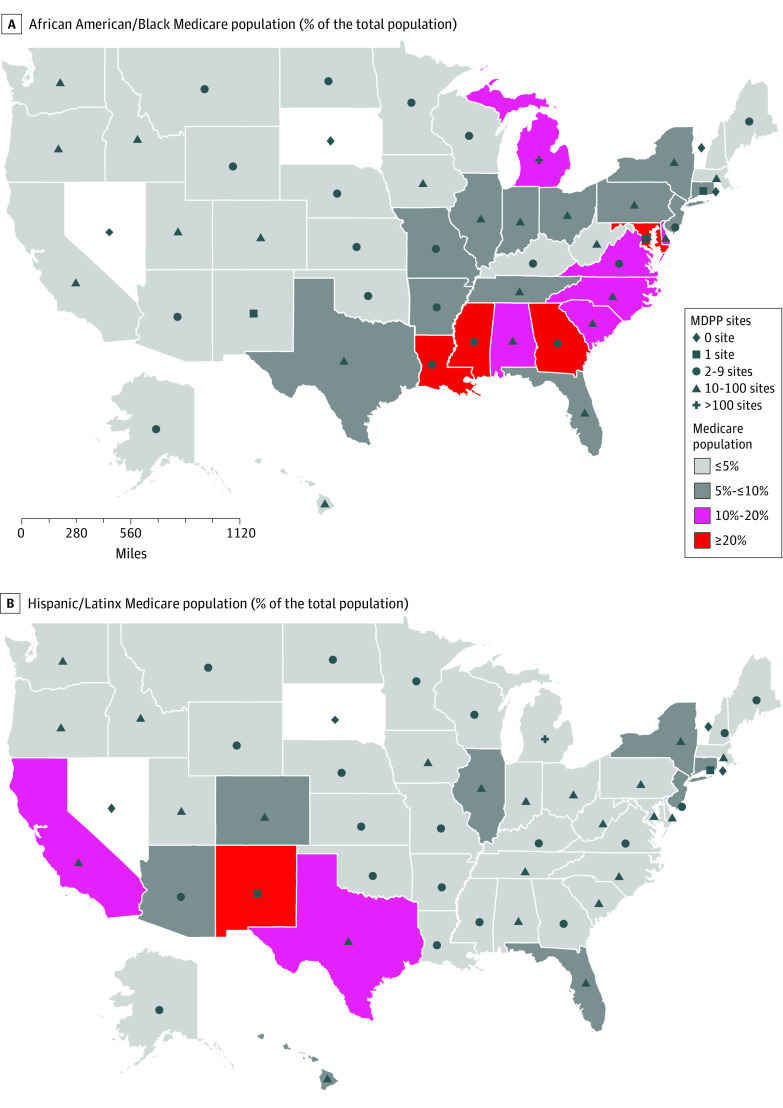
MDPP Supplier Site Distribution as of March 2021 and African American Medicare Population and Hispanic/Latinx Medicare Population at the State Level MDPP indicates Medicare Diabetes Prevention Program. The states in white (ie, Nevada, Rhode Island, South Dakota, and Vermont) indicate the states that did not have any MDPP sites as of March 2021.

## Discussion

Findings suggest that current MDPP suppliers, a proxy for accessibility, are not adequately meeting potential demand for services, especially for racial and ethnic minority groups. There is an urgent need to increase the availability and uptake of MDPP by expanding types of suppliers and encouraging innovations in service delivery, especially for those living in resource-poor areas. Rolling out the MDPP at the national level requires a systematic understanding of relationships among health care professionals, administrators, and health systems and the adoption of innovative implementation strategies. Such strategies could include adopting coordinated care practices, information exchange between systems and suppliers, and community partnerships. Study limitations include the inability to establish causal relationships due to the cross-sectional design, lack of uniform distribution of sites within or across states, which may not accurately reflect the actual situation in different parts of a state, and limited control for potentially confounding variables such as level of urbanization.
